# Endoscopic management of pseudo-lumen stapling following laparoscopic esophagojejunostomy: A case report

**DOI:** 10.1016/j.ijscr.2023.108830

**Published:** 2023-09-14

**Authors:** Seung Soo Lee

**Affiliations:** Department of Surgery, Kyungpook National University Hospital, School of Medicine, Kyungpook National University, Daegu, South Korea

**Keywords:** Case report, Endoscopy, Gastrectomy, Stomach neoplasms, Surgical staplers

## Abstract

**Introduction:**

Pseudo-lumen stapling can occur following an incidental submucosal introduction of a fork of the linear stapler into the esophageal side during esophagojejunostomy (EJS) after total gastrectomy. This leaves a mucosa-covered layer over the EJS site that can eventually cause an obstruction. If it is noticed intraoperatively, an immediate surgical take-down and repeat anastomosis might be chosen by most surgeons. However, these procedures might have side effects such as further dissection into the esophageal hiatus and unnecessary tension on the anastomosis. To our knowledge, no existing publication has presented a non-surgical management method for pseudo-lumen stapling.

**Presentation of case:**

A 64-year-old male underwent laparoscopic total gastrectomy with a pseudo-lumen stapling unnoticed during surgery. Upon its recognition on the third postoperative day, endoscopic release of the tissue covering the anastomosis was performed. The procedure was successful. Gastrographic examination on the sixth postoperative day confirmed a good passage of the contrast agent. Postoperative one-year endoscopic examination confirmed patent anastomosis without stenosis.

**Discussion:**

Although pseudo-lumen stapling is one of the most unwanted consequences of EJS using linear staplers, there is little information or documentation available as reference for cases encountered during clinical practice. This might be related to the tendency of surgeons to perform an immediate take-down, followed by repeat EJS when this is noticed during surgery. We were able to successfully overcome this problem without surgery following a series of early gastrographic and endoscopic procedures.

**Conclusion:**

Endoscopic release of the covering tissue should be considered a valid non-surgical solution to pseudo-lumen stapling.

## Highlights

• Submucosal insertion of a fork of the linear stapler leads to pseudo-lumen stapling.
• Pseudo-lumen stapling does not necessitate surgical take-down and re-anastomosis.
• Endoscopic release of covering tissue can be considered as a non-surgical option.

## 1 Introduction

Surgical staplers are commonly used to perform esophagojejunostomy (EJS) after total gastrectomy (TG) for gastric cancer [1,2]. According to surgeon's preference, both linear and circular staplers are used as frequently during laparoscopic TG, unlike general preference for circular staplers during open TG [[3], [4], [5]]. Which one is superior is a topic of ongoing debate. The use of a circular stapler during laparoscopic TG might allow surgeons to perform EJS in a manner somewhat similar to that of an open procedure. However, a circular stapler is bulkier than a linear stapler, making it difficult to establish secure intra-abdominal and intra-luminal accesses during a laparoscopic surgery.

**Fig. 1:**
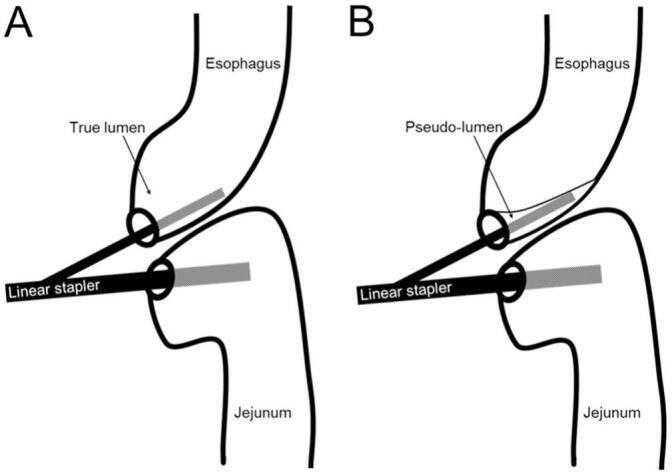
Esophago-jejunostomy using a linear stapler. (A) The fork on the esophageal end was inserted into the true lumen. (B) The fork on the esophageal end was inserted into the pseudo-lumen, resulting in a mucosa-covered layer over the anastomosis.

Although a linear stapler allows easy access through a conventional laparoscopic trocar, it is not without limitations. The surgeon needs to ensure a sufficient length of the abdominal esophagus for EJS using linear staplers [5,6]. Then sharp forks of the linear stapler must be inserted into each lumen of the esophageal and jejunal ends without causing injury. Pseudo-lumen stapling can occur following an incidental submucosal introduction of a fork of the linear stapler into the esophageal side [7,8]. Instead of introducing the fork into the true lumen of the esophagus, the sharp end can penetrate the submucosal layer and create a false submucosal lumen. After firing the stapler, a mucosal layer will cover the EJS site, eventually causing an obstruction (Fig. 1).

While pseudo-lumen stapling is one of the most unwanted consequences of EJS using linear staplers, there is little information or documentation available as reference for cases encountered during clinical practice. This might be related to the tendency of surgeons to perform an immediate take-down, followed by repeat EJS when this is noticed during surgery.

While an immediate take-down and repeat EJS might be the choice under normal circumstances, we experienced a case of pseudo-lumen stapling that went unnoticed during surgery and had to be managed non-surgically early after surgery. This case was reported according to SCARE 2020 criteria [9].

## 2 Case presentation

A 64-year-old male was diagnosed with stage 1 gastric cancer in the lesser curvature of the proximal body of the stomach. The cancer was asymptomatic. It was found on endoscopy during a regular health examination. The patient had no history of smoking, alcohol abuse, or previous illnesses except for a well-controlled hypertension. He had no prior surgeries or allergies. He had a body mass index of 27.9 kg/m^2^, which put him in the obese category based on the World Health Organization's definition of obesity for the Asia-Pacific region. He underwent a laparoscopic TG and D2 lymphadenectomy with curative intent. EJS was performed using linear surgical staplers. The length of the intra-abdominal esophagus was somewhat short. Extended dissection of the esophageal hiatus was performed to obtain an adequate length of the esophagus for EJS using linear staplers.

There was a slight degree of resistance upon insertion of the stapler fork into the esophageal end. It was decided that it could be pressure due to high level of insertion in the esophageal hiatus. After firing the stapler, the anastomosis migrated partially into the thorax. Direct visualization into the anastomosis was very limited. The rest of the surgical procedure was uneventful.

**Fig. 2:**
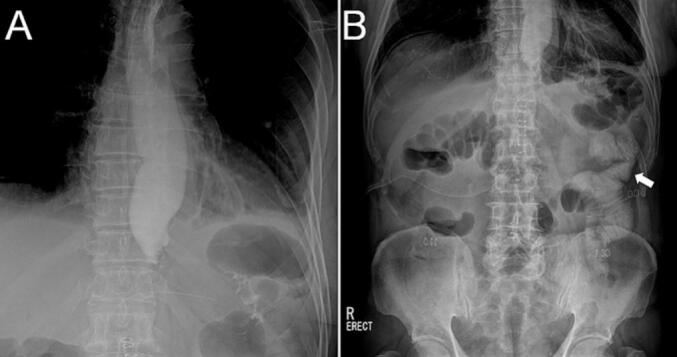
Gastrographic images taken on the third postoperative day. (A) The contrast did not pass beyond the anastomosis immediately after ingestion of a contrast medium. (B) Delayed passage of the contrast was observed after 1 h (single arrow), with a significant amount of the contrast remaining in the esophagus.

Gastrographic images were obtained on the third postoperative day before resuming a diet because resistance was felt during stapling. Initial gastrographic images taken seconds after the ingestion of a contrast medium showed no passage beyond the EJS site (Fig. 2). After an hour of ingestion, delayed passage of the contrast was revealed by an abdominal X-ray. However, there was still a significant amount of the contrast above the EJS site.

**Fig. 3:**
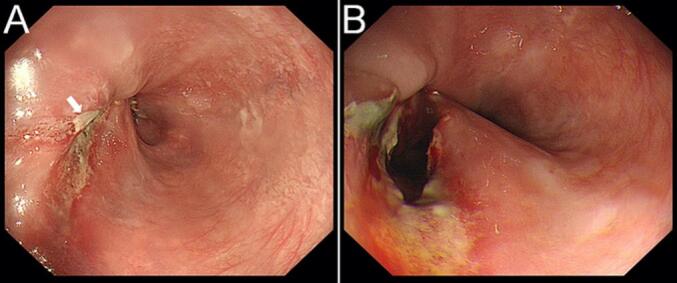
Endoscopic images taken on the third postoperative day. (A) A thin mucosa-covered layer with a tiny opening (single arrow) at the end over the anastomosis was noticed. (B) The jejunal side of the anastomosis was exposed following a gentle push of the scope over the covering tissue.

Upon immediate endoscopic examination, a thin mucosal layer with a tiny opening at the end was noticed over the EJS site (Fig. 3). The tissue layer was opened with a gentle push of the scope, exposing the jejunal side of the anastomosis. The patient was closely monitored after the procedure. His course was uneventful.

**Fig. 4:**
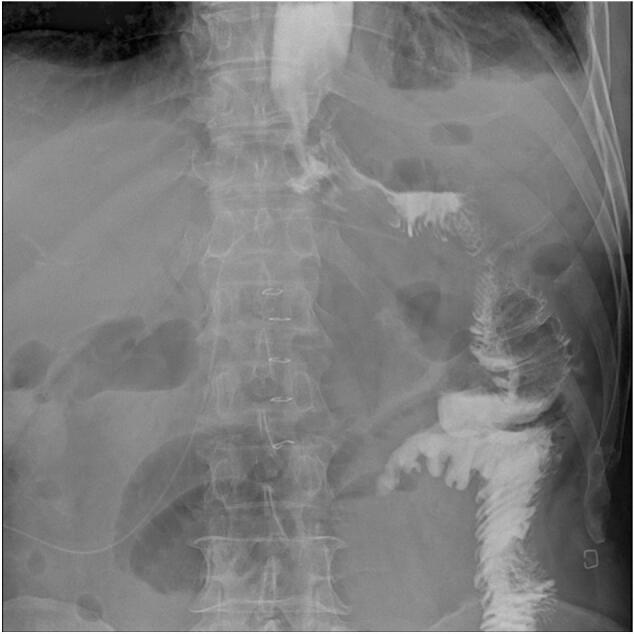
Gastrographic image taken on the sixth postoperative day. A good passage of the contrast agent was observed beyond the anastomosis upon ingestion.

On the sixth postoperative day, another gastrography was performed (Fig. 4) and a good passage of the contrast without leakage was confirmed. Oral intake was permitted thereafter. The patient was discharged at two weeks after surgery.

**Fig. 5:**
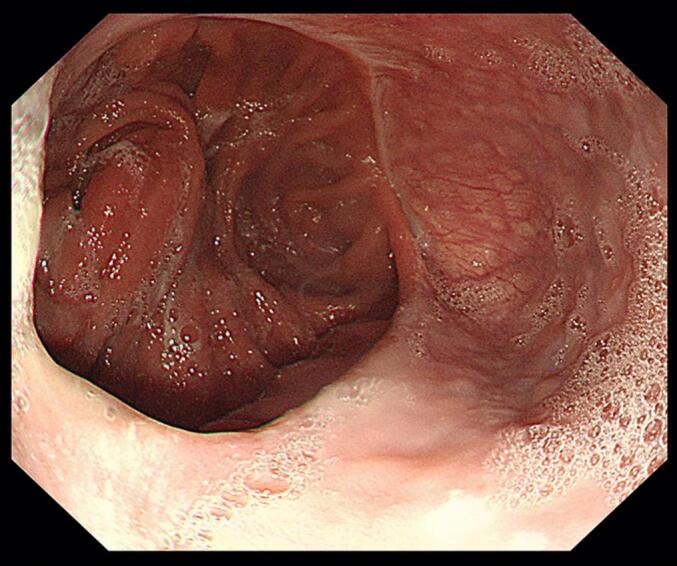
Endoscopic anastomosis finding one year after surgery. The anastomosis was patent without any sign of stenosis.

Histopathological examination of the surgical specimen revealed poorly differentiated adenocarcinoma with signet ring cells invading into the submucosal layer. There was no lymphovascular invasion. Both proximal and distal margins were negative for cancer cells. A total of 38 lymph nodes were retrieved. None of them was metastatic. The final stage was 1A according to the 8th edition of the Union for International Cancer Control (UICC) classification. The patient did not receive adjuvant chemotherapy. The patient did not complain of any significant gastrointestinal symptoms one year after surgery, and there was no sign of cancer recurrence. The endoscopic image taken at one year after surgery revealed a patent EJS without stenosis (Fig. 5).

## 3 Discussion

Pseudo-lumen stapling has been a big concern during EJS after laparoscopic TG. We were able to successfully overcome this problem without surgery by performing a series of early gastrographic and endoscopic procedures.

Although many surgeons are concerned with pseudo-lumen stapling, there has been little discussion on how to solve the problem. The discussion has mostly been about how to prevent it [7,10]. Full-thickness suturing of the esophageal wall or placing a nasogastric tube during EJS has been recommended to avoid pseudo-lumen stapling. However, these recommendations cannot guarantee that pseudo-lumen stapling does not occur, especially when the surgical field is very limited. We routinely perform full-thickness suturing of the esophageal wall. However, we have not been able to avoid this problem.

When uncertain about the non-surgical management of pseudo-lumen stapling, an immediate take-down and repeat EJS might be the only option for a surgeon after recognizing the condition. However, while doing so, the surgeon might have to conduct further dissection of the esophageal hiatus and accept increased tension at the anastomosis, which might lead to other adverse consequences [11].

Judging the anastomosis from a histopathological aspect, full mucosal, submucosal, muscularis propria, and adventitia layers of the esophagus are normally stapled against the jejunum after EJS. However, in the case of pseudo-lumen stapling, part of the submucosal layer and layers of the muscularis propria and adventitia are stapled against the jejunum. There are occasions when the mucosa and part of the submucosal layers of the gastrointestinal tract are removed for therapeutic purposes. Endoscopic mucosal dissection (ESD) is performed as a treatment for benign and selected malignant gastric tumors [12,13]. The gastric wall devoid of a mucosal layer has been shown to be capable of maintaining its integrity after ESD. ESD is not limited to the stomach. There are guidelines for performing ESD on the esophagus [[14], [15], [16]]. As such, the esophageal wall, might be strong enough to maintain EJS intact even after pseudo-lumen stapling.

The long-term clinical course is not without concerns, especially regarding stenosis caused by remnant mucosal and submucosal flaps at the anastomosis after the procedure. The postoperative one-year endoscopic examination of the present patient confirmed a patent EJS with neither stenosis nor remnant flaps.

The strength of this finding is that it broadens our treatment options following pseudo-lumen stapling. While the current finding does not preclude the necessity of surgical take-down or repeat EJS upon recognition of pseudo-lumen stapling, it provides sufficient evidence to reserve surgical take-down as a last resort. Even when it is recognized intraoperatively, intraoperative endoscopic release might become the first option. Endoscopic treatment has advantages over repeat EJS because it does not require further dissection into the esophageal hiatus or cause unnecessary tension on the anastomosis. This is the first report of a non-surgical management method of pseudo-lumen stapling. Findings of this study urge us to change our clinical approach toward pseudo-lumen stapling. In some cases, conventional approaches including surgical take-down and repeat anastomosis could be regarded as over-treatments.

Surgical take-down and repeat anastomosis should be considered upon detection of ongoing spillage or tearing of the staple line following pseudo-lumen stapling. An intraoperative endoscopic examination should be considered when pseudo-lumen stapling is suspected or detected while the staple line remains intact. Careful endoscopic release should be attempted when endoscopic examination confirms an intact anastomosis with a thin tissue layer covering it. Although the mucosa-covered layer over the EJS site in the present patient was thin enough to be opened by an unintended slightest push of the endoscope over the tissue, it is highly recommended to start the procedure with gentle pressure on the suspected area, perhaps with the small tip of endoscopic forceps. After confirming stability of the staple line, endoscopic release of the covering tissue should be attempted utilizing endoscopic electrocautery or knives, as necessary. An endoscopic release should be followed by a laparoscopic examination of the anastomosis. Anastomotic reinforcing sutures might help secure the anastomosis. Although an endoscopic management of pseudo-lumen stapling was followed in this patient by an early gastrographic examination (three days after the procedure) and a late endoscopic examination (one year after surgery), interim follow-up assessments within weeks after the procedure should help ensure reliability of the anastomosis. Of course, it is not our intention to devalue efforts for preventing pseudo-lumen stapling. It would be far more important to prevent the situation than to address it later.

## 4 Conclusion

Pseudo-lumen stapling does not always require surgical take-down or repeat EJS. Endoscopic release of covering tissue should be considered a valid non-surgical option for pseudo-lumen stapling. A strong suspicion, followed by gastrographic and endoscopic examination, could lead to early diagnosis and treatment.

## Sources of funding

This research did not receive any specific grant from funding agencies in the public, commercial, or not-for-profit sectors.

## Ethical approval

This study was approved by our Institutional Review Board (approval number: KNUH 2023–04-029).

## Registration of research studies

Not applicable.

## Consent

Written informed consent was obtained from the patient for publication and any accompanying images. A copy of the written consent is available for review by the Editor-in-Chief of this journal on request.

## CRediT authorship contribution statement

SSL made substantial contributions to all of the following: (1) the conception and design of the study, or acquisition of data, or analysis and interpretation of data, (2) drafting the article or revising it critically for important intellectual content, (3) final approval of the version to be submitted.

## Guarantor

Seung Soo Lee.

## Declaration of competing interest

There is no conflict of interest associated with this publication.
